# Feasibility, acceptability and cost‐effectiveness of a brief, lay counsellor‐delivered psychological treatment for men with alcohol dependence in primary care: an exploratory randomized controlled trial

**DOI:** 10.1111/add.14630

**Published:** 2019-05-11

**Authors:** Abhijit Nadkarni, Helen A. Weiss, Richard Velleman, Jim McCambridge, David McDaid, A‐La Park, Pratima Murthy, Benedict Weobong, Bhargav Bhat, Vikram Patel

**Affiliations:** ^1^ Sangath Socorro Goa India; ^2^ London School of Hygiene and Tropical Medicine London UK; ^3^ Institute of Psychiatry, Psychology, and Neuroscience King's College London London UK; ^4^ South London and Maudsley NHS Foundation Trust London UK; ^5^ University of Bath Bath UK; ^6^ University of York York UK; ^7^ Personal Social Services Research Unit, Department of Health Policy London School of Economics and Political Science London UK; ^8^ National Institute of Mental Health and Neurosciences Bengaluru India; ^9^ Harvard Medical School Boston MA USA

**Keywords:** Alcohol dependence, brief interventions, counselling for alcohol problems, India, lay counsellors, primary care

## Abstract

**Aims:**

To examine the feasibility, acceptability and preliminary cost‐effectiveness of a lay counsellor delivered psychological treatment for men with alcohol dependence in primary care.

**Design:**

Single‐blind individually randomized trial comparing counselling for alcohol problems (CAP) plus enhanced usual care (EUC) versus EUC only.

**Setting:**

Ten primary health centres in Goa, India.

**Participants:**

Men (*n* = 135) scoring ≥ 20 on the Alcohol Use Disorder Identification Test (AUDIT). Sixty‐six participants were randomized to EUC and 69 to CAP + EUC.

**Interventions:**

CAP, a lay counsellor‐delivered psychological treatment for harmful drinking, with referral to de‐addiction centre for medically assisted detoxification. EUC comprised consultation with physician, providing screening results and referral to a de‐addiction centre.

**Measurements:**

Baseline socio‐demographic data, readiness to change and perceived usefulness of counselling. Acceptability and feasibility process indicators such as data on screening and therapy. Outcomes were measured at 3 and 12 months post‐randomization and included remission, mean daily alcohol consumed, percentage of days abstinent (PDA), percentage of days of heavy drinking (PDHD), recovery, uptake of detoxification services, impacts of alcohol dependence, resource use and costs.

**Findings:**

Participants in the CAP + EUC arm had more numerically but not statistically significantly favourable outcomes compared with those in the EUC arm for (a) remission at 3 months [adjusted odds ratio (aOR) = 1.95, 95% confidence interval (CI) = 0.74–5.15] and 12 months (aOR = 1.90, 95% CI = 0.72–5.00), (b) proportion of non‐drinkers at 3 months (aOR = 1.26; 95% CI = 0.58–2.75) and 12 months (aOR = 1.25; 95% CI = 0.58–2.64) and (c) ethanol consumption among drinkers at 3 months (count ratio = 0.91; 95% CI = 0.58–1.45) and 12 months (count ratio = 1.06; 95% CI = 0.73–1.54). There was no statistically significant evidence of a difference in the occurrence of serious adverse events between the two arms. From a societal perspective, there was a 53% chance of CAP + EUC being cost‐effective in achieving remission at 12 months at the willingness‐to‐pay threshold of $415.

**Conclusions:**

Lay counsellor‐delivered psychological treatment for men with alcohol dependence (AD) in primary care may be effective in managing AD in low‐ and middle‐income countries. A definitive trial of the intervention is warranted.

## Introduction

Alcohol dependence (AD), a cluster of behavioural, cognitive and physiological phenomena in which alcohol use takes on a much higher priority for an individual than other behaviours, has been linked to a high level of disability and economic burden and an elevated risk of mortality compared to the general population 1, 2, 3, 4, 5, 6. In India, although there has been a rapid change in patterns and trends of alcohol use in recent years, alcohol consumption remains a predominantly male activity, characterized by the frequent and heavy drinking of spirits 7, 8. Further, in India, 21% of the adult general population drinks alcohol, with 17–26% of them estimated to be alcohol‐dependent, i.e. approximately 4% of the general population 7.

Despite the existence of effective treatment options for AD, the treatment gap for all forms of harmful drinking globally remains high (78%), especially in low‐ and middle‐income countries (LMICs) including India, where the recent National Mental Health Survey reported a treatment gap of 86% 9, 10, 11.

Access to care is limited, due to both patient‐related factors (e.g. attitudes, knowledge), systemic barriers (e.g. availability, affordability, provider skills and knowledge) and contextual factors (e.g. stigma) 12, 13. One way to overcome the health system barriers, especially in resource‐poor settings, is to deliver interventions through task sharing (i.e. rational redistribution of tasks among health work‐force teams) using non‐specialist health workers (NSHW) to overcome the shortage of specialist human resources. There is growing evidence supporting the effectiveness of NSHW‐delivered interventions for alcohol use disorders (AUD), including in LMICs such as Thailand, Kenya and India 14, 15, 16, 17, 18. However, these interventions were designed to target hazardous and harmful drinking, not alcohol dependence. Finally, although there is extensive evidence supporting the efficacy of brief interventions (BI) among people with non‐dependent AUD, there is a lack of evidence that BIs are effective for people with AD 19, 20, and it is standard practice for those with AD to be referred for treatment in specialist services.

PREMIUM (Program for Effective Mental Health Interventions in Under‐Resourced Health Systems) is a research programme which aimed to develop scalable psychological treatments that are culturally appropriate, affordable and feasible for delivery by NSHWs, including for harmful drinking [counselling for alcohol problems (CAP)] 21. A definitive randomized controlled trial (RCT) demonstrated the cost‐effectiveness of CAP delivered by NSHWs for harmful drinkers [defined by the Alcohol Use Disorder Identification Test (AUDIT) score of 12–19] in routine primary health‐care settings in India 17, 18.

This paper describes the findings of an exploratory trial conducted in parallel with the larger RCT to examine the following: (a) feasibility of identifying and recruiting men with probable AD in primary care, (b) feasibility of delivering a brief treatment for AD by lay counsellors in primary care, (c) acceptability and safety of the treatment and (d) preliminary cost‐effectiveness of the treatment on engagement with specialist services and drinking and associated outcomes.

## Methods

The methods for PREMIUM are fully described in the trial protocol (ISRCTN76465238) and publications regarding the trial of CAP in harmful drinkers 17, 18, 22. The trial was approved by the Institutional Review Boards of the London School of Hygiene and Tropical Medicine, Sangath (the implementing institution in India) and the Indian Council of Medical Research.

### Setting

Goa is in western India (1.4 million population). Alcohol is easily available in Goa, at cheap rates due to lower excise duties. Goa has higher prevalence of drinking in men (39% in the community, 59% in primary care and 69% in industrial workers) compared to most parts of India, and has a high prevalence of AUDs (15% of men in primary care) 23, 24, 25.

### Study design and participants

A parallel‐arm single‐blind individually randomized controlled trial (RCT) was conducted in 10 primary health centres (PHCs). Attenders at the PHCs were screened with the AUDIT 26 if they were 18–65‐year‐old males (females were not screened, as prevalence of any alcohol use in women is very low in India), residing in the PHC catchment area, intending to reside at the same address for at least 12 months, able to communicate clearly, not presenting with an emergency medical condition and able to comprehend one of the programme's four languages. Probable dependent drinkers, defined as scoring ≥ 20 on the AUDIT who provided informed consent, were recruited into the study. The AUDIT is a 10‐item screening questionnaire developed by the World Health Organization (WHO) for the detection of AUD, and has been validated in India 27. While a score of ≥ 20 is not conclusive evidence of dependence, this cut‐off is in line with expert guidance on the use of this instrument, although dependence has been idenitfied in primary‐care populations at lower scores 28. A randomization list in randomly sized blocks (two to four), stratified by PHC, was generated by a statistician independent of the trial. The randomization code was concealed and consenting participants were randomized at the individual level by trained health assistants based at the primary health centres in a 1 : 1 allocation scheme to either of two intervention arms [enhanced usual care (EUC) or EUC plus CAP] after completion of the baseline assessments, using sequentially numbered opaque sealed envelopes. Enrolment continued until the required sample size for harmful drinkers for the definitive RCT described above was achieved, and was conducted between 28 October 2013 and 29 July 2015; the final 12‐month assessment was completed on 30 August 2016. Physicians providing EUC were masked to allocation status, as were the independent assessors who performed the outcome assessments, and these people had no contact with the PHCs or other team members. All authors, apart from the data manager (B.B.), were masked.

### Sample size estimations

The sample size for this exploratory RCT was not informed a priori by formal sample size calculations. Enrolment for this exploratory RCT was based on achieving the required sample size for harmful drinkers for the definitive RCT. The achieved sample size was judged to be adequate to answer the descriptive primary questions about acceptability and feasibility.

### Interventions

EUC followed a contextualized version of the WHO Mental Health Gap Action Programme (mhGAP) guidelines 29 and comprised consultation with the PHC physician and provision of the screening results to the patient, with the primary action to be taken by the PHC physician being referral to the local de‐addiction centre.

CAP is a manualized psychological treatment delivered in three phases over a maximum of four sessions (each lasting approximately 30–45 minutes) at weekly to fortnightly intervals. The initial phase involves detailed assessment followed by personalized feedback; the middle phase involves helping the patient to develop cognitive and behavioural skills and techniques and the ending phase involves the patient learning how to manage potential or actual relapses using the skills acquired in the middle phase. Referral to the local secondary or tertiary care de‐addiction centre for medically assisted detoxification consisted of informing the participants about the need for detoxification, providing them with details about de‐addiction centres and suggesting that they attend. Detoxification services in Goa are delivered in out‐ and in‐patient settings in the public (two district hospitals and one tertiary care psychiatry teaching institute) and private (rehabilitation centres) sectors.

The approach adopted by the CAP counsellor is informed by motivational interviewing (MI). The same counsellors who delivered CAP to harmful drinkers 17, 18 delivered the intervention to the dependent drinkers. The 11 counsellors were adults with no prior professional training and/or qualification in the field of mental health. They had completed at least high school education, were fluent in the vernacular languages used in the study settings and were trained and supervised in delivering CAP through a rigorous process. Further details of the intervention and of the selection, training and supervision of the counsellors are described elsewhere 21, 30. The intervention content and related training material can be accessed on‐line (http://nextgenu.org/course/view.php?id=167#0 and http://www.sangath.in/evidence‐based‐intervention‐manuals/).

### Data

#### Baseline socio‐demographic data

Baseline socio‐demographic data. Readiness to change (not at all, a little ready, somewhat ready, moderately ready, already trying to change) and perceived usefulness of counselling (no, a little, somewhat useful, moderately useful, very useful) were rated on a Likert scale and analysed as binary variables (not at all to little ready versus somewhat ready to already trying; and no to somewhat useful versus moderately to very useful).

#### Acceptability and feasibility process indicators

Acceptability and feasibility process indicators were collected through the course of the trial. These included data on screening, therapy (e.g. number of sessions, duration of sessions, planned discharge and referrals) and safety (serious adverse events). A participant was classified as a ‘planned discharge’ if at least one of the following criteria were met: treatment completion was decided in collaboration with the counsellor, treatment goals were achieved or the maximum of four sessions were completed. The serious adverse events (SAEs) measured included death due to any cause during the past 12 months, unplanned hospitalization during the past 12 months and suicidal behaviour (suicidal thoughts in past 14 days and/or suicidal attempts in past 3 months) at 3‐ and 12‐month outcome evaluation.

#### Effectiveness outcomes

Effectiveness outcomes were measured at 3 and 12 months post‐randomization. The two primary drinking outcomes were remission defined as an AUDIT score < 8, and mean daily alcohol (in grams pure ethanol) in the past 14 days immediately preceding the outcome evaluation. The secondary drinking outcomes include percentage of days abstinent (PDA); percentage days of heavy drinking (PDHD); and recovery (AUDIT < 8 at both 3 and 12 months). The mean daily alcohol consumption, PDA and PDHD were generated from the time‐line follow‐back (TLFB), a calendar tool supplemented by memory aids to obtain retrospective estimates of daily drinking over a specified time‐period 31. Other secondary outcomes include uptake of detoxification services and impacts of alcohol dependence, i.e. (a) short inventory of problems (SIP), a 15‐item questionnaire which assesses physical, social, intrapersonal, impulsive and interpersonal consequences of alcohol consumption, a higher score indicating greater adverse impacts (range 0–15) 32; (b) depression measured using the Patient Health Questionnaire‐9 (PHQ‐9), a nine‐item questionnaire of depressive symptoms assessed on a scale of 0–3 (range 0–27) 33; (c) World Health Organization Disability Assessment Schedule (WHODAS), a 12‐item questionnaire for measuring functional impairment during the previous 30 days; a higher score indicating greater disability (range 0–48) 34; (d) total days unable to work; (e) suicidal behaviour; and (f) interpersonal violence. In a joint meeting of the Trial Steering Committee and Data Monitoring and Safety Committee before unblinding, two additional outcomes (PDA and PDHD generated from the TLFB) were added to bring the trial into line with recommendations of the National Institute on Alcohol Abuse and Alcoholism (NIAAA).

#### Resource use and costs

Resource use and costs were estimated using a modified version of the Client Service Receipt Inventory 35. We used information concerning contact with the counsellor to estimate CAP delivery costs, which took into account training, supervision and salary costs.

### Statistical analyses

Acceptability and feasibility data were analysed using descriptive statistics; wherever appropriate, comparisons were made using *t*‐test and one‐way analysis of variance (ANOVA) and *χ*^2^ test for continuous and categorical outcomes, respectively, and logistic regression was used to calculate odds ratios (OR) for predictors of dropout. Given the highly skewed distribution of ethanol consumption and small sample size in this trial, multiple imputation (MI) was problematic. Hence, considering recent methodological developments which indicate that for trials with one primary end‐point, analyses which adjust for factors associated with missingness are equivalent to MI 36, to handle missing data we followed the analyses strategy of adjusting for baseline variables associated with drop‐out. Zero‐inflated negative binomial (ZINB) regression 37 was used to estimate the intervention effect for positively skewed over‐dispersed outcomes with an excess of zeros, i.e. for the mean daily alcohol consumption and total number of days unable to work. Other continuous outcomes (with normally distributed residuals) were analysed using linear regression and binary outcomes were analysed using binary logistic regression. All models were adjusted for PHC as a fixed‐effect to allow for within‐PHC clustering and for baseline AUDIT score. For ZINB regression, the intervention effect is estimated for all participants in one model as an adjusted odds ratio (aOR) with 95% confidence interval (CI) for proportion with zero (i.e. no reported drinking) and adjusted count ratio among those with non‐zero responses, respectively. For example, in the case of ratio of mean amount consumed between those in the intervention versus control arm, we used the ZINB regression to estimate the probability of abstinence among all participants, and the mean amount consumed, only among those who did drink. For other continuous outcomes, the intervention effect was reported as the adjusted mean difference (AMD) and for binary outcomes the intervention effect was reported as aOR. Sensitivity analyses for linear and logistic regression models included adjustment for counsellor as a random‐effect. For the remission outcome we conducted a ‘worst case scenario’ sensitivity analysis, in which we assumed that all individuals who dropped out reverted to their pre‐intervention behaviour, i.e. baseline AUDIT score. Besides effectiveness analyses separately for the 3‐ and 12‐month time‐points, we also conducted repeated‐measures analysis, including analysis of change over time within each of the end‐points. The repeated‐measures analysis included a treatment × time interaction term to allow for a different intervention effect at 3 versus 12 months. We conducted a per‐protocol analysis which included only those participants who had a planned discharge. We compared differences in mean costs between the two arms using standard parametric tests. We imputed missing values and bootstrapped incremental costs effectiveness ratios (ICERs) to derive 95% CIs. We explored statistical uncertainty concerning the ICERs through cost‐effectiveness acceptability curves showing the likelihood that CAP would be cost‐effective at different levels of willingness‐to‐pay thresholds. All costs are presented in 2015 international dollars. Statistical analyses were conducted using STATA versions 14 and 15.

## Results

### Acceptability and feasibility

#### Trial recruitment and retention

Between October 28, 2013, and July 29, 2015, 16 007 (21.7%) of the 73 887 adult male PHC attenders assessed met the eligibility criteria for screening, and of these, 14 773 were screened using the AUDIT. Of the screened participants, 206 (1.4%) were eligible (AUDIT score ≥ 20) for inclusion in this exploratory trial, and 135 (65.5%) consented to participate and were enrolled.

**Figure 1:**
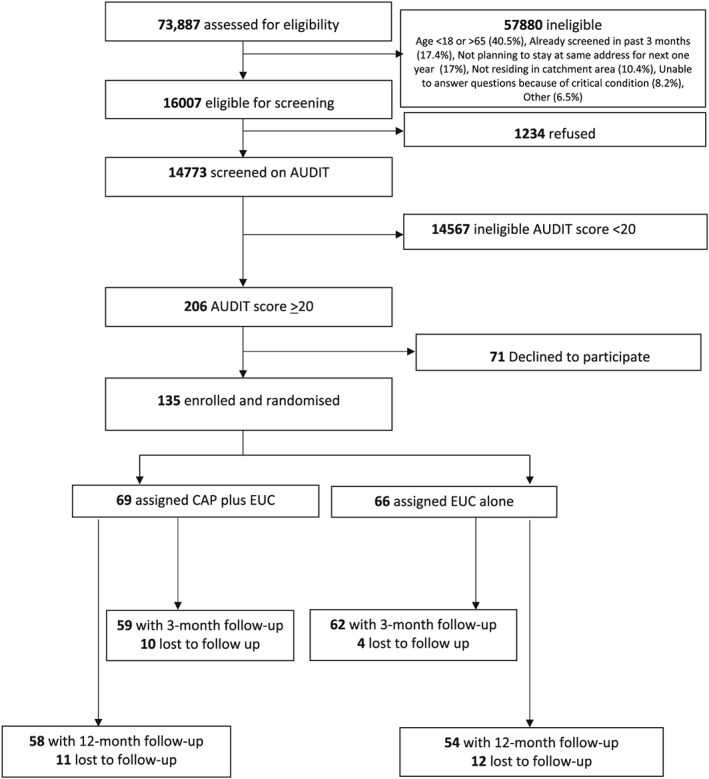
Counselling for alcohol problems trial flow‐chart. CAP = counselling for alcohol problems; EUC = enhanced usual care; AUDIT = Alcohol Use Disorders Identification Test

A total of 66 participants were randomized to EUC and 69 to CAP plus EUC (Fig. 1). Baseline characteristics were similar by arm, with the exception of those in the CAP plus EUC arm being slightly older and having lower expectations of usefulness of counselling (Table 1).

**Table 1:** Baseline characteristics of the trial participants by arm.

	CAP arm (n = 69)	EUC arm (n = 66)
Mean age in years (SD)	43.3 (11.5)	39.7 (10.4)
Marital status (*n*, %)		
Married	51 (73.9)	54 (81.8)
Never married/separated/divorced/widowed	18 (26.1)	12 (18.2)
Occupation (n, %)		
Unemployed	12 (17.4)	10 (15.2)
Employed	57 (82.7)	56 (84.8)
Education (*n*, %)		
No formal education	12 (17.4)	11 (16.7)
Completed primary education	39 (56.5)	40 (60.6)
Completed secondary education or higher	18 (26.0)	15 (22.7)
Patient's expectation of usefulness of counselling (*n*, %)		
A little/somewhat useful	10 (14.5)	13 (19.7)
Moderately useful	23 (33.3)	8 (12.1)
Very useful	36 (52.2)	45 (68.2)
Mean AUDIT score (SD)	23.9 (3.6)	24.7 (4.1)
Readiness to change (*n*, %)		
Not at all to little ready	8 (11.6)	11 (16.7)
Somewhat ready to already trying	61 (88.4)	55 (83.3)

AUDIT = Alcohol Use Disorder Identification Test; CAP = Counselling for Alcohol Problems; EUC = enhanced usual care; SD = standard deviation.

Of the 135 recruited participants, 121 (89.6%) completed outcomes at the 3‐month post‐treatment end‐point and 112 (83.0%) at 12‐month follow‐up. AUDIT scores for both 3‐ and 12‐month end‐points were available for 107 participants (79.3%). On multivariable analysis at 3 months, greater expectation of usefulness of counselling was associated with dropout from the study (OR = 6.53, 95% CI = 1.50–28.41; *P* = 0.01); and at 12 months, older age (OR = 1.07; 95% CI = 1.00–1.13; *P* = 0.04) and greater readiness to change (OR = 3.76; 95% CI = 1.12–12.56; *P* = 0.03) was associated with dropout from the study (Supporting information, Table S1). These variables were included in multivariable regression models for effectiveness at 3 and 12 months, respectively.

#### Engagement with treatment

Overall, 16 (23.2%) participants completed all four sessions, 18 (26.1%) completed only three sessions, 13 (18.8%) completed only two sessions and 22 (31.9%) participants completed only one session. The mean number of sessions completed was 2.4 (SD = 1.2). The mean session duration was 45.9 (SD = 9.6) minutes, with a range of 26.7–67.0 minutes. Of the 47 participants assigned homework, 33 (70.2%) completed or attempted it between sessions. There was no association between number of sessions completed and duration of sessions or involvement of significant other (SO).

Overall, 40 (58.0%) participants had a planned discharge from treatment. There were no statistically significant differences in baseline characteristics between those who had unplanned versus planned discharge, apart from those with an unplanned discharge being younger than those with a planned discharge (mean age 39.6, SD = 11.1 versus 46.0, SD = 11.1; *P* = 0.02). There was no significant association between indicators of treatment engagement (number of sessions attended, planned discharge) with drinking outcomes in the CAP plus EUC arm (Supporting information, Tables S2 and S3). There was no association between planned discharge and involvement of SO, but planned discharge was significantly associated with shorter mean duration of sessions [51.78, SD = 10.05 versus 42.43 (7.52), *P* = 0.0001] (Supporting information, Table S3).

#### Acceptability of specific intervention strategies

We compared acceptability and feasibility indicators (described below) between AD in this feasibility trial and other harmful drinkers in the definitive CAP trial. The mean duration of sessions was slightly greater for AD than other harmful drinkers [45.9 (9.6) versus 42.4 (9.4); *P* = 0.01], but there were no other significant differences (Table 2).

**Table 2:** Comparison of acceptability and feasibility indicators.

	CAP for harmful drinkers in parallel PREMIUM trial (n = 188)	CAP for dependent drinkers in this PREMIUM trial (n = 69)	P
Mean number of sessions (SD)	2.4 (1.1)	2.4 (1.2)	0.84
Mean duration of sessions in minutes (SD)	42.4 (9.4)	45.9 (9.6)	0.01
Homework completion^a^ *n* (%)	102 (76.7)	33 (70.2)	0.38
Planned discharge *n* (%)	131 (69.7)	40 (58.0)	0.08
Significant other (SO) involvement^b^ *n* (%)			
Session 2	23 (17.4)	7 (15.6)	0.77
Session 3	11 (13.4)	1 (2.9)	0.09
Session 4	0 (0)	0 (0)	

CAP = counselling for alcohol problems; SD = standard deviation.

a Among those assigned homework;

b among those who attended the session.

Participants were requested to invite one SO (e.g. spouse, sibling, close friend) to attend sessions. SOs of seven (10.1%) participants attended at least one session. Referral data for detoxification were available only for the CAP arm. Of the 69 participants in this arm, 23 (33.3%) did not consent for referral to detoxification services at all during the course of the treatment and the rest were referred at least once. Those who did not consent for referral for detoxification received fewer sessions than those who were referred [mean = 1.9 (1.0) versus 2.8 (1.2), *P* = 0.002]. However, none of the participants in the trial reported any contact with detoxification services at 3‐ and 12‐month outcome evaluation. There was no significant difference in drinking and other outcomes when compared between those who were referred and those who did not consent for referral to detoxification.

### Effectiveness

**Figure 2:**
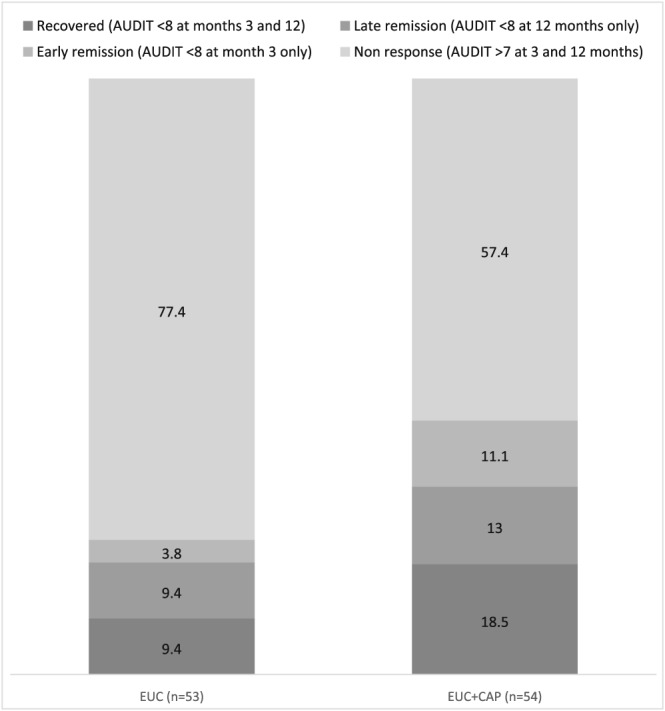
Clinical outcomes in participants with 3‐ and 12‐month Alcohol Use Disorders Identification Test (AUDIT) data (n = 107)

Tables 3 and 4 describe the outcomes at 3 and 12 months, respectively. There was no significant difference between the arms for (a) proportion with remission at 3 months (27.1 versus 14.5%; aOR = 1.95, 95% CI = 0.74–5.15, *P* = 0.18) and 12 months (31.0 versus 18.5%; aOR = 1.90, 95% CI = 0.72–5.00, *P* = 0.19); (b) proportion of participants reporting no alcohol consumption in the past 14 days at 3 months (35.6 versus 30.7%; aOR = 1.26 95% CI = 0.58–2.75; *P* = 0.57) and 12 months (34.5 versus 29.6%; aOR = 1.25 95% CI = 0.58–2.64; *P* = 0.57); and (c) consumption among those who reported any drinking in this period at 3 months (58.9 g, SD = 60.0 versus 59.2 g, SD = 59.5; count ratio = 0.91, 95% CI = 0.58–1.45, *P* = 0.70) and 12 months (45.2 g, SD = 29.0 versus 60.4 g, SD = 50.1; count ratio = 1.06, 95% CI = 0.73–1.54, *P* = 0.77). For the ‘worst case scenario’ sensitivity analysis there was no significant difference between the two arms for proportion with remission at 3 months (23.2 versus 13.6%; aOR = 1.67, 95% CI = 0.64–4.32; *P* = 0.29) and 12 months (26.1 versus 15.2%; aOR = 1.78, 95% CI = 0.71–4.45; *P* = 0.22). For the secondary outcomes, some of the estimated effects were large, including PDA at 3 months, PDHD at 12 months and recovery. In addition, at 12 months, there were fewer days heavy drinking, lower PHQ‐9 score, lower WHO‐DAS score and fewer days of inability to work among participants in favour of the CAP arm. Compared to the EUC arm, a greater proportion in the intervention arm experienced an early as well as late remission, and had recovered; in contrast, a greater proportion in the EUC arm remained dependent drinkers at both end‐points (Fig. 2). However, these findings were not statistically significant. After adjusting for counsellor as a random effect, there was a significant intervention effect on PDHD (AMD –11.4; 95% CI = –21.6 to –1.2; *P* = 0.03) and WHO‐DAS score (AMD –3.2; 95% CI = –6.1 to –0.3; *P* = 0.03) at 12 months (Supporting information, Table S4). In the per‐protocol analysis, the participants in the CAP arm had significantly lower PDHD (AMD –18.1, 95% CI = 31.5 to –4.7, *P* = 0.009) and WHO‐DAS scores (AMD –4.5, 95% CI = –8.0 to –1.0, *P* = 0.01) at 12 months. The remaining outcomes favoured CAP, but did not reach statistical significance (Supporting information, Tables S5 and S6). A significant proportion of participants in the CAP arm experienced ‘any response’ (early/late remission or recovery) compared to EUC (42.6 versus 22.6%, *P* = 0.03). Repeated‐measures analyses showed no significant interaction with time (3 or 12 months) for alcohol consumption in the past 14 days, amount of drinking among drinkers or remission, suggesting that there was no evidence that the effect of the intervention changed over time. There was no significant difference in the number of participants who experienced SAEs between the two arms (see Table 5). Eleven participants had an unplanned hospitalization once, and three participants had unplanned hospitalization events twice; 17 and 20 participants, respectively, reported suicidal behaviour once at 3 and 12 months.

**Table 3:** Effects of the CAP plus EUC compared with EUC alone on clinical and other outcomes at 3 months.

Outcome	EUC + CAP^a^ (n = 59)	EUC^a^ (n = 62)	Intervention effect (95% CI)^b^	P
Primary outcomes				
Remission (AUDIT < 8) (*n*, %)	16 (27.1)	9 (14.5)	aOR 1.95 (0.74–5.15)	0.18
Daily standard ethanol consumed in the past 14 days^c^				
Non‐drinkers (*n*, %)	21 (35.6)	19 (30.7)	aOR 1.26 (0.58–2.75)	0.57
Ethanol consumption (g) among drinkers (mean, SD)	58.9 (60.0)	59.2 (59.5)	Count ratio 0.91 (0.58–1.45)	0.70
Secondary outcomes				
Percentage of days abstinent (PDA) (mean %, SD)^f^	60.7 (42.1)	50.2 (41.8)	AMD 9.4 (−6.5–25.2)	0.24
Percentage days of heavy drinking (PDHD) (mean %, SD)^f^	20.5 (35.5)	22.0 (36.5)	AMD −2.2 (−15.8–11.4)	0.75
Patient Health Questionnaire‐9 (PHQ‐9) (mean, SD)	6.9 (6.2)	7.4 (6.0)	AMD −0.5 (−2.8–1.7)	0.63
Suicidal behaviour (*n*, %)^d^	9 (15.3)	8 (12.9)	aOR 1.21 (0.41–3.63)	0.73
Short inventory of problems (SIP) (mean, SD)	14.8 (12.5)	17.1 (10.5)	AMD −2.6 (−6.6–1.5)	0.21
WHO‐DAS score (mean, SD)	5.8 (7.6)	6.7 (6.5)	AMD −1.1 (−3.7–1.5)	0.40
Days unable to work^c^				
None (*n*, %)	31 (52.5)	31 (50.0)	aOR 1.12 (0.53–2.34)	0.77
Days unable to work when ≥ 1 day reported (mean, SD)	12.1 (10.8)	11.3 (10.5)	Count ratio 1.0 (0.66–1.52)	0.99
Perpetration of intimate partner violence^e^ (*n*, %)	8 (16.0)	8 (17.8)	aOR 1.08 (0.32–3.59)	0.90

aOR = adjusted odds ratio; AMD = adjusted mean difference; AUDIT = Alcohol Use Disorder Identification Test; CAP = counselling for alcohol problems; CI = confidence interval; EUC = enhanced usual care; g = grams; SD = standard deviation; WHO‐DAS = WHO disability assessment schedule.

a Among those with observed data at 3 months.

b Complete case adjusted for adjusted for PHC as a fixed effect, baseline AUDIT score, and expectation from treatment.

c Analysed with a zero‐inflated negative binomial model which fits two parameters in one model, i.e. the proportion with response of zero (e.g. no drinking in 14 days; or no days unable to work), and the mean count (e.g. ethanol consumption or days unable to work) among people with a non‐zero (positive) response.

d Suicidal thoughts during the past 2 weeks were assessed through the relevant PHQ‐9 item while suicide attempts were assessed during the 3‐month period leading up to the outcome follow‐up assessment.

e Among married participants only.

f Not previously specified in trials protocol, but specified in published analysis plan.

**Table 4:** Effects of the CAP plus EUC compared with EUC alone on clinical outcomes and other outcomes at 12 months.

Outcome	EUC + CAP^a^ (n = 58)	EUC^a^ (n = 54)	Intervention effect (95% CI)^b^	P
Primary outcomes				
Remission (AUDIT < 8) (*n*, %)	18 (31.0)	10 (18.5)	aOR 1.90 (0.72–5.00)	0.19
Daily standard ethanol consumed in the past 14 days^c^				
Non‐drinkers (*n*, %)	20 (34.5)	16 (29.6)	aOR 1.25 (0.58–2.64)	0.57
Ethanol consumption (g) among drinkers (Mean [SD])	45.2 (29.0)	60.4 (50.1)	Count Ratio 1.06 (0.73–1.54)	0.77
Secondary outcomes				
Recovery (AUDIT < 8 at 3 and 12 months (*n*, %)^e^	10 (18.5)	5 (9.4)	aOR 1.91 (0.52–7.01)	0.33
Percent of days abstinent (PDA) (mean %, SD)^e^	56.8 (42.5)	53.2 (40.3)	AMD 0.9 (−15.9–17.6)	0.92
Percentage days of heavy drinking (PDHD) (mean %, SD)^e^	10.3 (22.4)	23.4 (33.1)	AMD −9.9 (−20.9–1.1)	0.08
Patient Health Questionnaire‐9 (PHQ‐9) (mean, SD)	6.1 (6.3)	7.9 (6.7)	AMD −1.2 (−3.8–1.4)	0.37
Suicidal behaviour (*n*, %)^f^	9 (15.5)	11 (20.4)	aOR 0.87 (0.29–2.60)	0.81
Short inventory of problems (SIP) (mean, SD)	12.7 (12.0)	16.5 (11.0)	AMD −2.8 (−7.3–1.7)	0.21
WHO‐DAS score (mean, SD)	4.8 (7.4)	8.1 (8.3)	AMD −2.7 (−5.8–0.5)	0.09
Days unable to work^c^				
None (*n*, %)	35 (60.3)	26 (48.2)	aOR 1.63 (0.75–3.56)	0.22
Days unable to work when ≥1 day reported (mean, SD)	13.7 (11.6)	12.3 (10.5)	Count ratio 1.04 (0.61–1.75)	0.89
Perpetration of intimate partner violence^d^ (*n*, %)	5 (11.4)	6 (13.6)	aOR 5.67 (0.71–45.04)	0.10

aOR = adjusted odds ratio; AMD = adjusted mean difference; AUDIT = Alcohol Use Disorder Identification Test; CAP = counselling for alcohol problems; CI = confidence interval; EUC = enhanced usual care; g = grams; SD = standard deviation; WHO‐DAS=WHO Disability Assessment Schedule.

a Among those with observed data at 12 months.

b Complete case adjusted for adjusted for PHC as a fixed effect, baseline AUDIT score, age and readiness to change at baseline.

c Analysed with a zero‐inflated negative binomial model which fits two parameters in one model, i.e. the proportion with response of zero (e.g. no drinking in 14 days; or no days unable to work), and the mean count (e.g. ethanol consumption or days unable to work) among people with a non‐zero (positive) response.

d Among married participants only.

e Not previously specified in trials protocol, but specified in published analysis plan,

f Suicidal thoughts during the past 2 weeks were assessed through the relevant PHQ‐9 item while suicide attempts were assessed during the 3‐month period leading up to the outcome follow‐up assessment.

**Table 5:** Number (%) of participants who experienced serious adverse events.

	CAP n (%)	EUC n (%)	P
Unplanned hospitalization in past 12 months (*n* = 112)	6 (10.3)	8 (14.8)	0.48
Death in past 12 months (*n* = 135)	0 (0)	1 (1.52)	0.31
Suicidal behaviour at 3 months (*n* = 121)	9 (15.3)	8 (12.9)	0.71
Suicidal behaviour at 12 months (*n* = 112)	9 (15.5)	11 (20.4)	0.50

CAP = counselling for alcohol problems; EUC = enhanced usual care.

### Costs

Overall, there is no significant difference either in health service costs or in wider societal costs between the two arms. Compared to EUC, health‐care costs in the CAP arm are higher in all categories, but productivity costs linked to work cutback and work loss are lower in the CAP arm (Table 6). From a health‐care perspective, there is a 20% chance of CAP being cost‐effective at the willingness‐to‐pay threshold of $415 (equivalent to 1 month's wages for an unskilled manual worker in Goa). However, from a societal perspective, there is a 53% chance of CAP being cost‐effective (Supporting information, Figs S1 and S2).

**Table 6:** Mean costs (2015 international dollars) per person in EUC + CAP and EUC groups over 12 months.

Type of cost	EUC + CAP (n = 69)	EUC (n = 66)	Mean difference (95% CI)	P
CAP intervention costs				
CAP intervention (SE)	39.93 (5.10)	0 (0)	39.93 (29.75, 50.11)	< 0.001
Health service utilization				
PHC doctor consultations (SE)	38.26 (7.22)	28.64 (4.70)	9.63 (−7.48, 26.68)	0.27
Hospital doctor consultations (SE)	34.11 (6.90)	9.20 (2.66)	24.91 (10.23, 39.59)	0.001
Detoxification services	8.53 (4.70)	1.72 (1.06)	6.81 (−2.79, 16.40)	0.16
Hospital admissions (SE)	85.06 (44.33)	71.90 (29.51)	13.16 (−92.31, 118.63)	0.81
Laboratory tests (SE)	21.14 (6.88)	11.48 (2.31)	9.66 (−4.78, 24.10)	0.187
Medicines (SE)	22.22 (7.87)	12.02 (3.04)	10.20 (−6.57, 26.97)	0.23
Total health service utilization Costs (SE)	209.33 (53.73)	134.96 (34.69)	74.36 (−52.33, 201.05)	0.25
Total health system costs				
Total health system costs (SE)	249.26 (53.24)	134.96 (34.69)	114.29 (−11.56, 240.53)	0.08
Productivity costs				
Time costs to service users and families (SE)	230.37 (69.98)	184.64 (47.04)	45.72 (−121.25, 212.70)	0.59
Productivity losses (SE)	348.37 (48.48)	469.96 (57.62)	−121.59 (−270.58, 27.41)	0.11
Total societal costs				
Societal perspective (SE)	828.00 (140.94)	789.56 (94.00)	38.43 (−297.05, 373.93)	0.821

SE = standard error; CAP = counselling for alcohol problems; CI = confidence interval; EUC = enhanced usual care.

## Discussion

This exploratory study has observed that it is feasible to identify and recruit men with probable AD in primary‐care facilities in Goa, that it is feasible for lay counsellors to safely deliver the CAP, a brief psychosocial intervention for these patients, that this is acceptable to the target group and that there were better, but statistically non‐significant, outcomes in the CAP arm.

Furthermore, our process indicators suggest that it is feasible for lay counsellors to identify men with probable AD through universal screening in primary care and retain a reasonable proportion of them in treatment and deliver at least two sessions of counselling. The study participants mainly chose abstinence as an appropriate treatment goal; most engaged with strategies such as completion of homework between sessions and consented for the involvement of their family members in treatment. These process indicators show a pattern similar to those in the definitive RCT with harmful drinkers.

There was no evidence of increased referral to detoxification, indicating low acceptability of the prevailing facility‐based tertiary care as it is offered in India. This finding is consistent with evidence that brief alcohol interventions by themselves do not lead to increased access to specialist alcohol treatment services 38. For low resource settings, a more efficient utilization of resources would be treatment of AD in community settings, through programmes based on the principle of collaborative care. Such programmes have proven effects in improving clinical outcomes, cost‐effectiveness and acceptability and overcome challenges related to accessibility and acceptability of treatment 39, 40.

In the definitive RCT of CAP in harmful drinkers in which this exploratory trial was nested, the intervention was shown to be effective in increasing remission, abstinence and percentage of days abstinent at 3 months; and at 12 months follow‐up there was a sustained effect of the intervention on these outcomes 17, 18. The effectiveness findings of this exploratory trial are broadly consistent with the results of the definitive RCT. As expected for an exploratory trial, there were few statistically significant intervention effects although, for most outcomes, participants in the CAP arm had more favourable outcomes compared with those in the EUC arm. Finally, although the higher health‐care costs (indicative of more contact with services as a result of CAP) mitigated the effects of the lower productivity costs in the intervention arm, the probability of the intervention being cost‐effective over 12 months exceeded 50%.

Our findings suggest the potential applicability of CAP for the management of AD in low resource settings. The most likely reason for the absence of statistically significant differences is the limited power of this study, leading to low precision of the estimates of effect. It is also possible that AD requires a more intensive psychosocial treatment, and a brief treatment such as the CAP might not be sufficient to deal with the complex cognitive and behavioural processes associated with AD. If this is the case, then supplementing CAP with other strategies could be more effective in improving drinking outcomes in AD. Such strategies could possibly include discussions to address barriers to accessing care and concerns about treatment efficacy, education about available pharmacological treatments and supplementing therapy with more intensive efforts, such as telephone monitoring and collaborative case management.

Besides the lack of power to examine effectiveness, our study had several other limitations. The outcomes were reliant upon self‐report data, which are susceptible to social desirability bias. This might lead to under‐reporting on self‐reports of alcohol consumption and its harmful consequences, which could be differential between trial arms 19. However, in the absence of more objective and sensitive measures, self‐report is accepted as the most reliable method for assessment of drinking outcomes in alcohol treatment trials 41. Our findings cannot be generalized to women, as we tested CAP only in men; and the generalizibility of our findings to other states of India and elsewhere will need further exploration. As we have tested multiple hypotheses, there are chances of false‐positives. However, it is not unusual to use multiple outcome measures in feasibility trials, as one of the goals of such trials is to identify and test appropriate outcome measures for a definitive trial. The low prevalence rate of alcohol dependence in our study might be the result of the stigma associated with alcohol dependence which hinders help‐seeking and could promote socially desirable responses to the screening and outcome tools. However, such low detection and recruitment rates are not unusual in trials involving participants with substance use disorders 42, and feasibility trials are helpful in identifying such potential barriers and developing suitable mitigation strategies for the definitive trial (e.g. non‐monetary incentives). The strengths of our trial lie in its rigorous implementation procedures and the lack of intensive assessments at baseline, as assessment reactivity has been found to be problematic in alcohol use disorder trials 43. Finally, the evaluation of outcomes at 3 and 12 months allowed us to examine not just the immediate effects of CAP but also whether these effects were sustained over a relatively longer period, as this is critical for a disorder that is highly prone to relapse and recurrence.

The evidence base for treatment of AD is predominantly derived from high‐income countries and concerns psychosocial interventions delivered by highly trained health professionals in specialist treatment settings 44, 45. Thus, CAP is unique, as it is designed to be delivered by lay counsellors in primary‐care settings. This makes it potentially scalable in low resource settings. While there is no evidence for efficacy of brief interventions among those with very heavy alcohol use or alcohol dependence 20, a definitive trial of the CAP is warranted by our findings of its feasibility, acceptability and effectiveness. However, such a trial would need to address the challenges we faced in this exploratory study. A definitive trial would need a sample size of 520 and 386 to detect the difference in the primary outcome of remission we observed in this trial, at 90 and 80% power, respectively, 5% level of significance and allowing for 17% loss to follow‐up. Attaining such a sample size will require more and diverse recruitment sites; for example, secondary and tertiary clinics for people with alcohol‐related medical disorders. If effective, such an intervention could position CAP as a first‐line psychosocial intervention for the full range of AUDs in primary care.

## Clinical trial registration

ISRCTN76465238 (http://www.isrctn.com/ISRCTN76465238)

## Declaration of interests

None.

## Supporting information


**Table S1** Comparison of participants who were followed up and LTFU at 3 months and 12 months
AUDIT = Alcohol Use Disorder Identification Test, SD = Standard deviation
**Table S2** Association of treatment engagement (number of sessions completed) with acceptability/feasibility indicators and drinking outcomes in the CAP arm of the trial
**Table S3** Association of treatment engagement (planned discharge and completion of homework) with drinking outcomes in the CAP arm of the trial
**Table S4** Intervention effect on outcomes at 3 and 12 months (random effects)
**Table S5** Effects of the CAP plus EUC compared with EUC alone on clinical and other outcomes at 3 months (Per protocol analyses)
**Table S6** Effects of the CAP plus EUC compared with EUC alone on clinical outcomes and other outcomes at 12 months (Per protocol analyses)
**Figure S1** Cost‐effectiveness acceptability curve: Willingness to pay per remission achieved via CAP from a health system perspective
**Figure S2** Cost‐effectiveness acceptability curve: Willingness to pay per remission achieved via CAP from a health system perspective.
