# Supplementary material for: Feasibility, acceptability and cost‐effectiveness of a brief, lay counsellor‐delivered psychological treatment for men with alcohol dependence in primary care: an exploratory randomized controlled trial
Source: Addiction. 2019 May 11;114(7):1192–203. doi: 10.1111/add.14630 (PMC6563185; doi:10.1111/add.14630)
Supplement: Supplementary file 1 — Table S1 Comparison of participants who were followed up and LTFU at 3 months and 12 months AUDIT = Alcohol Use Disorder Identification Test, SD = Standard deviation Table S2 Association of treatment engagement (number of sessions completed) with acceptability/feasibility indicators and drinking outcomes in the CAP arm of the trial Table S3 Association of treatment engagement (planned discharge and completion of homework) with drinking outcomes in the CAP arm of the trial Table S4 Intervention effect on outcomes at 3 and 12 months (random effects) Table S5 Effects of the CAP plus EUC compared with EUC alone on clinical and other outcomes at 3 months (Per protocol analyses) Table S6 Effects of the CAP plus EUC compared with EUC alone on clinical outcomes and other outcomes at 12 months (Per protocol analyses) Figure S1 Cost‐effectiveness acceptability curve: Willingness to pay per remission achieved via CAP from a health system perspective Figure S2 Cost‐effectiveness acceptability curve: Willingness to pay per remission achieved via CAP from a health system perspective. [file ADD-114-1192-s001.doc]

**Supplementary Figure 1. Cost-effectiveness acceptability curve: Willingness to pay per remission achieved via CAP from a health system perspective**


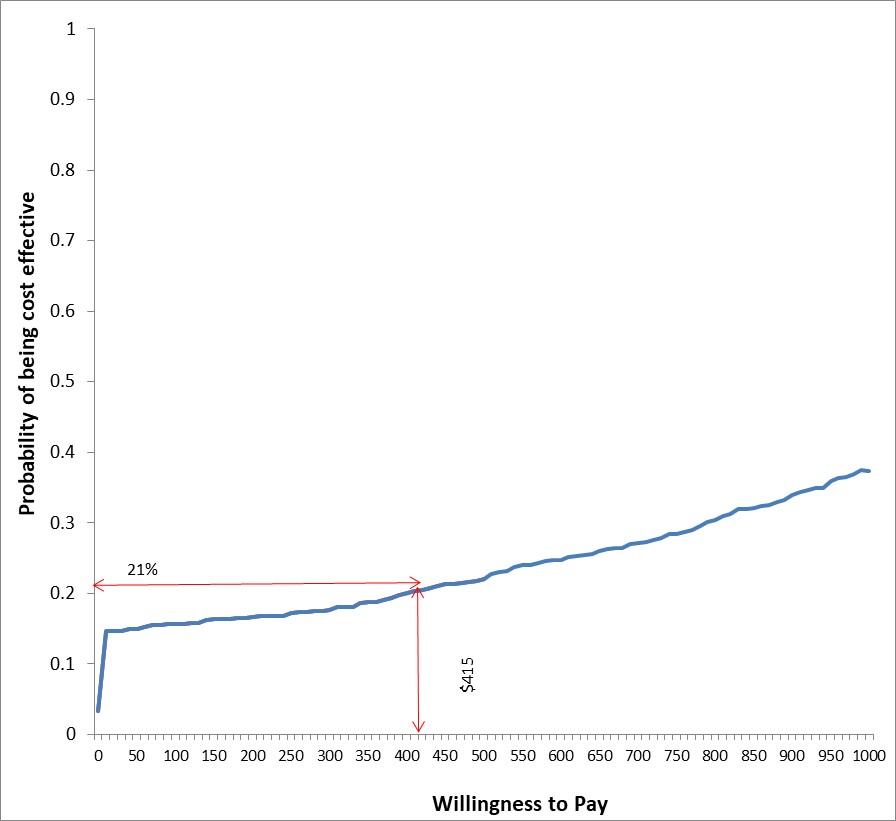


**Supplementary Figure 2. Cost-effectiveness acceptability curve: Willingness to pay per remission achieved via CAP from a health system perspective**
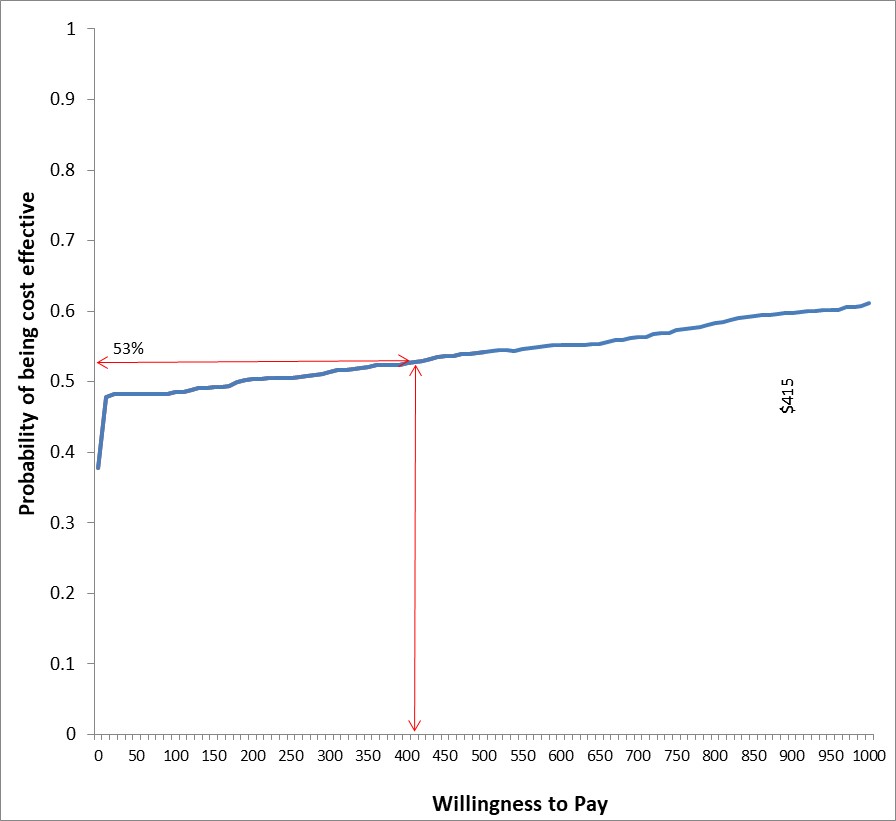


**Supplementary T**able 1: Comparison of participants who were followed up and LTFU at 3 months and 12 months

|  | **Lost before 3 month evaluation**  **(n=14; 10.4%)** | **Completed 3 month outcome evaluation (n=121; 89.6%)** | **p** | **Lost before 12 month evaluation**  **(n=23; 17%)** | **Completed 12 month outcome evaluation (n=112; 83.0%)** | **p** |
| --- | --- | --- | --- | --- | --- | --- |
| Age (years) (mean [SD]) | 32.5 (8.0) | 42.6 (10.9) | 0.001 | 35.7 (12.4) | 42.8 (10.4) | 0.005 |
| **Marital status** (n [%])  Married  Never married/Separated/Divorced/Widower | 9 (64.3)  5 (35.7) | 98 (81.0)  23 (19.0) | 0.17 | 16 (69.6)  7 (30.4) | 91 (81.3)  21 (18.8) | 0.26 |
| **Occupation** (n [%])  Unemployed  Employed | 0 (0)  14 (100.0) | 22 (18.2)  99 (81.8) | 0.13 | 2 (8.7)  21 (91.3) | 20 (17.9)  92 (82.1) | 0.37 |
| **Education** (n [%])  No formal education  Completed primary and above | 1 (7.1)  13 (92.9) | 22 (18.2)  99 (81.8) | 0.46 | 3 (13.0)  20 (87.0) | 20 (17.9)  92 (82.1) | 0.76 |
| **Patient’s expectation of counselling** (n [%])  No/a little/somewhat useful  Moderately or very useful | 6 (42.9)  8 (57.1) | 17 (14.1)  104 (86.0) | 0.02 | 7 (30.4)  16 (69.6) | 16 (14.3)  96 (85.7) | 0.07 |
| **Readiness to change** (n[%])  Not at all to little ready  Somewhat ready to already trying | 3 (21.4)  11 (78.6) | 16 (13.2)  105 (86.8) | 0.42 | 7 (30.4)  16 (69.6) | 12 (10.7)  100 (89.3) | 0.02 |
| **AUDIT score** (mean (SD)) | 24.9 (4.0) | 24.2 (3.9) | 0.56 | 25.6 (4.2) | 24.0 (3.8) | 0.08 |

AUDIT=Alcohol Use Disorder Identification Test, SD=Standard deviation

Supplementary Table 2: Association of treatment engagement (number of sessions completed) with acceptability/feasibility indicators and drinking outcomes in the CAP arm of the trial

| **Outcome** | **Completed one session n=22 (31.9%)** | **Completed two sessions n=13 (18.8%)** | **Completed three sessions n=18 (26.1%)** | **Completed four sessions 16 (23.2%)** | **p** |
| --- | --- | --- | --- | --- | --- |
| **Acceptability/feasibility indicators** |  |  |  |  |  |
| Mean duration of sessions in minutes (SD) | 54.53 (8.39) | 47.31 (8.77) | 39.65 (7.27) | 42.78 (7.03) | 0.81 |
| Significant Other (SO) involvement^2^ n(%) | 0 | 2 | 5 | 1 | 0.25 |
| **Drinking outcomes** |  |  |  |  |  |
| Remission (AUDIT<8) (n) at 3 months | 2 | 4 | 4 | 6 | 0.28 |
| Percent of days abstinent [mean% (SD)]) at 3 months | 41.6 (44.4) | 68.2 (40.5) | 58.5 (41.9) | 79.0 (34.5) | 0.08 |
| Percent days of heavy drinking [mean% (SD)]) at 3 months | 37.4 (43.9) | 13.6 (29.2) | 12.1 (25.7) | 15.2 (34.8) | 0.14 |
| Remission (AUDIT<8) (n) at 12 months | 4 | 3 | 6 | 5 | 0.63 |
| Percent of days abstinent [mean% (SD)]) at 12 months | 23.5 (34.4) | 42.0 (34.2) | 37.3 (34.0) | 41.8 (41.7) | 0.57 |
| Percent days of heavy drinking [mean% (SD)]) at 12 months | 16.4 (32.8) | 10.4 (21.9) | 7.1 (13.8) | 6.0 (12.3) | 0.58 |
| Recovery (AUDIT<8 at 3 and 12 months (n) | 2 | 2 | 2 | 4 | 0.61 |

^1^Amongst those assigned homework; ^2^Among those who attended the session

AUDIT=Alcohol Use Disorder Identification Test, SD=Standard deviation

**Supplementary Table 3: Association of treatment engagement (planned discharge and completion of homework) with drinking outcomes in the CAP arm of the trial**

|  | **Dropout from treatment**  **N=29 (42.0%)** | **Planned discharge**  **N=40 (58.0%)** | **p** |
| --- | --- | --- | --- |
| **Acceptability/feasibility indicators** |  |  |  |
| Mean duration of sessions in minutes (SD) | 51.78 (10.05) | 42.43 (7.52) | 0.0001 |
| Significant Other (SO) involvement^2^ n(%) | 1 | 7 | 0.69 |
| **Drinking outcomes** |  |  |  |
| Remission (AUDIT<8) (n) at 3 months | 5 | 11 | 0.46 |
| Percent of days abstinent [mean% (SD)]) at 3 months | 48.4 (43.1) | 68.5 (40.1) | 0.07 |
| Percent days of heavy drinking [mean% (SD)]) at 3 months | 31.7 (42.7) | 13.3 (28.4) | 0.05 |
| Remission (AUDIT<8) (n) at 12 months | 5 | 13 | 0.16 |
| Percent of days abstinent [mean% (SD)]) at 12 months | 29.8 (35.2) | 37.9 (35.7) | 0.49 |
| Percent days of heavy drinking [mean% (SD)]) at 12 months | 16.7 (31.0) | 5.9 (12.0) | 0.07 |
| Recovery (AUDIT<8 at 3 and 12 months (n) | 3 | 7 | 0.52 |

AUDIT=Alcohol Use Disorder Identification Test, SD=Standard deviation

Supplementary Table 4: Intervention effect on outcomes at 3 and 12 months (random effects)

| **Outcome** | **EUC+CAP^1^ (n=59)** | **EUC^1^ (n=62)** | **Intervention effect (95% CI) at 3 months** | **p** | **EUC+CAP^1^ (n=58)** | **EUC^1^ (n=54)** | **Intervention effect (95% CI) at 12 months** | **p** |
| --- | --- | --- | --- | --- | --- | --- | --- | --- |
| **PRIMARY OUTCOMES** |  |  |  |  |  |  |  |  |
| Remission (AUDIT<8) [n(%)] | 16 (27.1) | 9 (14.5) | aOR 1.94 (0.74-5.15) | 0.18 | 18 (31.0) | 10 (18.5) | aOR 1.90 (0.72-4.97) | 0.19 |
| **SECONDARY OUTCOMES** |  |  |  |  |  |  |  |  |
| Percent of days abstinent [Mean%(SD)] | 60.7 (42.1) | 50.2 (41.8) | AMD 9.4 (-5.6-24.3) | 0.22 | 56.8 (42.5) | 53.2 (40.3) | AMD 0.8 (-14.8-16.5) | 0.92 |
| Percent days of heavy drinking [Mean%(SD)] | 20.5 (35.5) | 22.0 (36.5) | AMD -2.4 (-15.2-10.5) | 0.72 | 10.3 (22.4) | 23.4 (33.1) | AMD -11.4 (-21.6- -1.2) | 0.03 |
| Short inventory of problems (SIP) [Mean(SD)] | 14.8 (12.5) | 17.1 (10.5) | AMD -1.8 (-13.3-9.6) | 0.75 | 12.7 (12.0) | 16.5 (11.0) | AMD -3.5 (-7.7-0.7) | 0.09 |
| Patient Health Questionnaire-9 (PHQ-9) [Mean(SD)] | 6.9 (6.2) | 7.4 (6.0) | AMD -0.4 (-5.9-5.1) | 0.88 | 6.1 (6.3) | 7.9 (6.7) | AMD -1.4 (-3.8-1.0) | 0.24 |
| WHO-DAS score [Mean(SD)] | 5.8 (7.6) | 6.7 (6.5) | AMD -1.1 (-3.6-1.3) | 0.37 | 4.8 (7.4) | 8.1 (8.3) | AMD -3.2 (-6.1- -0.3) | 0.03 |
| Perpetration of intimate partner violence [n(%)] | 8 (16.0) | 8 (17.8) | aOR 1.13 (0.34-3.71) | 0.84 | 5 (11.4) | 6 (13.6) | aOR 2.02 (0.40-10.19) | 0.39 |

^1^Among those with observed data at 3 months ^2^Among those with observed data at 12 months

aOR=Adjusted Odds Ratio, AMD=Adjusted Mean Difference, AUDIT=Alcohol Use Disorder Identification Test, CI=Confidence Interval, WHO-DAS=WHO Disability Assessment Schedule

**Supplementary table 5.** Effects of the CAP plus EUC compared with EUC alone on clinical and other outcomes at 3 months (Per protocol analyses)

| **Outcome** | **EUC+CAP^1^ (n=40)** | **EUC^1^ (n=66)** | **Intervention effect (95% CI)^2^** | **p** |
| --- | --- | --- | --- | --- |
| Remission (AUDIT<8) (n [%]) | 11 (30.6) | 9 (14.5) | aOR 2.63 (0.78-8.89) | 0.12 |
| Daily standard ethanol consumed in the past 14 days^3^ |  |  |  |  |
| -Non-drinkers (n [%]) | 16 (44.4) | 19 (30.7) | aOR 1.80 (0.76-4.31) | 0.18 |
| -Ethanol consumption (g) among drinkers (Mean [SD]) | 43.3 (40.9) | 59.2 (59.5) | Count Ratio 0.74 (0.44- 1.27) | 0.28 |
| Percent of days abstinent [mean% (SD)])* | 68.5 (6.7) | 50.2 (41.8) | AMD 16.5 (-3.1-36.1) | 0.10 |
| Percent days of heavy drinking [mean% (SD)])* | 13.3 (4.7) | 22.0 (36.5) | AMD -8.7 (-24.7-7.3) | 0.28 |
| Patient Health Questionnaire-9 (PHQ-9) [mean (SD)] | 5.4 (5.6) | 7.4 (6.0) | AMD -1.6 (-4.2-1.1) | 0.25 |
| Suicidal behaviour (n [%])^#^ | 3 (8.3) | 8 (12.9) | aOR 0.91 (0.18-4.44) | 0.90 |
| Short inventory of problems (SIP) (mean (SD)) | 12.6 (11.8) | 17.1 (10.5) | AMD -2.8 (-7.6-1.9) | 0.24 |
| WHO-DAS score (mean (SD)) | 5.1 (6.0) | 6.7 (6.5) | AMD -1.6 (-4.4-1.3) | 0.28 |
| Days unable to work^3^ |  |  |  |  |
| - None (n [%]) | 19 (52.8) | 31 (50.0) | aOR 1.16 (0.49-2.72) | 0.74 |
| - Days unable to work when >1 day reported (mean (SD)) | 12.9 (10.1) | 12.7 (10.2) | Count ratio 0.92 (0.56-1.51) | 0.74 |
| Perpetration of intimate partner violence^4^ (n [%]) | 3 (10.3) | 8 (16.0) | aOR 1.54 (0.27-8.89) | 0.63 |

^1^Among those with observed data at 3 months ^2^Complete case adjusted for adjusted for PHC as a fixed effect, baseline AUDIT score, patient’s age at baseline, and expectation from treatment  ^3^Analysed with a zero-inflated negative binomial model which fits two parameters in one model i.e. the proportion with response of zero (e.g. no drinking in 14 days; or no days unable to work), and the mean count (e.g. ethanol consumption or days unable to work) among people with a non-zero (positive) response ^4^Among married participants only *Not previously specified in trials protocol but specified in published analysis plan ^#^Suicidal thoughts over the past two weeks were assessed through the relevant PHQ-9 item while suicide attempts were assessed over the 3-month period leading up to the outcome follow up assessment

**Supplementary table 6.** Effects of the CAP plus EUC compared with EUC alone on clinical outcomes and other outcomes at 12 months (Per protocol analyses)

| **Outcome** | | **EUC+CAP^1^ (n=58)** | **EUC^1^ (n=54)** | **Intervention effect (95% CI)^2^** | **p** |
| --- | --- | --- | --- | --- | --- |
| Remission (AUDIT<8) (n [%]) | | 13 (38.2) | 10 (18.5) | aOR 3.14 (0.97-10.15) | 0.06 |
| Daily standard ethanol consumed in the past 14 days^3^ | |  |  |  |  |
| -Non-drinkers (n [%]) | | 14 (41.2) | 16 (29.6) | aOR 1.58 (0.67-3.74) | 0.29 |
| -Ethanol consumption (g) among drinkers (Mean [SD]) | | 37.2 (22.4) | 60.4 (50.1) | Count Ratio 0.95 (0.58- 1.55) | 0.84 |
| Recovery (AUDIT<8 at 3 and 12 months (n [%])* | | 7 (21.2) | 5 (9.4) | aOR 2.65 (0.54-13.01) | 0.23 |
| Percent of days abstinent [mean% (SD)])* | | 63.4 (41.2) | 53.2 (40.3) | AMD 9.1 (-11.1-29.3) | 0.37 |
| Percent days of heavy drinking [mean% (SD)])* | | 5.9 (2.1) | 23.4 (33.1) | AMD -18.1 (-31.5- -4.7) | 0.009 |
| Patient Health Questionnaire-9 (PHQ-9) [mean (SD)] | | 5.1 (5.5) | 7.9 (6.7) | AMD -2.4 (-5.5-0.6) | 0.11 |
|  | Suicidal behaviour (n [%])^#^ | 4 (11.8) | 11 (20.4) | aOR 0.75 (0.17-3.30) | 0.71 |
|  | Short inventory of problems (SIP) (mean (SD)) | 11.4 (11.4) | 16.5 (11.0) | AMD -3.9 (-9.1-1.24) | 0.13 |
|  | WHO-DAS score (mean (SD)) | 3.3 (5.3) | 8.1 (8.3) | AMD -4.5 (-8.0- -1.0) | 0.01 |
|  | Days unable to work^3^ |  |  |  |  |
|  | - None (n [%]) | 18 (52.9) | 26 (48.2) | aOR 1.21 (0.49-3.03) | 0.67 |
|  | - Days unable to work when >1 day reported (mean (SD)) | 11.6 (11.5) | 12.3 (10.5) | Count ratio 0.83 (0.44-1.57) | 0.57 |
|  | Perpetration of intimate partner violence^4^ (n [%]) | 2 (7.1) | 6 (13.6) | aOR 2.53 (0.17-36.8) | 0.50 |

^1^Among those with observed data at 12 months ^2^Complete case adjusted for adjusted for PHC as a fixed effect, baseline AUDIT score, and patient’s age at baseline  ^3^Analysed with a zero-inflated negative binomial model which fits two parameters in one model i.e. the proportion with response of zero (e.g. no drinking in 14 days; or no days unable to work), and the mean count (e.g. ethanol consumption or days unable to work) among people with a non-zero (positive) response ^4^Among married participants only *Not previously specified in trials protocol but specified in published analysis plan ^#^Suicidal thoughts over the past two weeks were assessed through the relevant PHQ-9 item while suicide attempts were assessed over the 3-month period leading up to the outcome follow up assessment
